# Beta-Lactam Probability of Target Attainment Success: Cefepime as a Case Study

**DOI:** 10.3390/antibiotics12030444

**Published:** 2023-02-23

**Authors:** Daniel J. Selig, Adrian T. Kress, Robert J. Nadeau, Jesse P. DeLuca

**Affiliations:** Walter Reed Army Institute of Research, Experimental Therapeutics Branch, Silver Spring, MD 20910, USA

**Keywords:** antibiotics, beta-lactams, cefepime, cephalosporins, population pharmacokinetics, probability of target attainment, simulations

## Abstract

Introduction: Probability of target attainment (PTA) analysis using Monte Carlo simulations has become a mainstay of dose optimization. We highlight the technical and clinical factors that may affect PTA for beta-lactams. Methods: We performed a mini review in adults to explore factors relating to cefepime PTA success and how researchers incorporate PTA into dosing decisions. In addition, we investigated, via simulations with a population pharmacokinetic (PK) model, factors that may affect cefepime PTA success. Results: The mini review included 14 articles. PTA results were generally consistent, given the differences in patient populations. However, dosing recommendations were more varied and appeared to depend on the definition of pharmacodynamic (PD) target, definition of PTA success and specific clinical considerations. Only 3 of 14 articles performed formal toxicological analysis. Simulations demonstrated that the largest determinants of cefepime PTA were the choice of PD target, continuous vs. intermittent infusion and creatinine clearance. Assumptions for protein binding, steady state vs. first dose, and simulating different sampling schemes may impact PTA success under certain conditions. The choice of one or two compartments had a minimal effect on PTA. Conclusions: PTA results may be similar with different assumptions and techniques. However, dose recommendation may differ significantly based on the selection of PD target, definition of PTA success and considerations specific to a patient population. Demographics and the PK parameters used to simulate time-concentration profiles should be derived from patient data applicable to the purpose of the PTA. There should be strong clinical rationale for dose selection. When possible, safety and toxicity should be considered in addition to PTA success.

## 1. Introduction

Population pharmacokinetic (PK) modeling is a common technique to estimate the mean and variability of PK parameters within a patient population [1]. Clinical data such as creatinine clearance (CrCl), weight, age, etc., may be incorporated into such models as covariates, and help to explain the variability of drug PK models in subsets of patients within the population of interest [2]. Once a population PK model is validated, the model may be used to simulate dosing regimens to help determine an optimal dose based on a given exposure-response metric and variability within the population. This methodology is part of a model-informed drug development approach, which has been formally recognized by the Food and Drug Administration (FDA) [3].

For antibiotic drug development or dose optimization in special populations, simulations with population PK models in conjunction with a pharmacodynamic (PD) target forms the basis of probability of target attainment (PTA). Three minimum inhibitory concentration (MIC)-based PD indices are commonly used to inform antibiotic dose optimization: (1) time free drug is above MIC (fT% > MIC), (2) ratio of free drug area under the curve (fAUC) to MIC (fAUC:MIC), and (3) ratio of drug maximum concentration (C_max_) to MIC (C_max_:MIC) (PMC5039113). Classically, fT% > MIC is the optimal metric for beta-lactam antibiotics, where fAUC:MIC and C_max_:MIC are utilized for fluoroquinolones and aminoglycosides, respectively [4].

The general method for a PTA analysis begins by defining a PTA target. Commonly defined targets for beta-lactams are 40%, 50%, and 100% fT% > 1–8 X MIC. Although the choice of target is somewhat arbitrary and is dependent on the patient population, there remains a debate around selecting the optimal target [5,6,7]. Once a target is chosen, a validated population PK model is used to simulate thousands of virtual patient PK profiles. The percent of simulated PK profiles that achieve the pre-defined metric is called the PTA [8]. The PTA is considered successful for a particular dosing regimen if PTA is >80–90% [9,10,11]. However, the decision is again arbitrary, and the optimal choice of success may depend on the patient population, specific infection, overall risk benefit ratio and local resources.

Generally, although many decisions in performing PTA have a component of subjectivity, most researchers using PTA to aid dose optimization are guided by the same underlying principles. However, the process of choosing a PD metric for PTA analysis may lead to drastically different dosing conclusions based on the chosen target [7]. Furthermore, there are other less commonly used metrics, such as a cumulative fraction of response (CFR), which aid in the decision of which population subgroups to simulate, and other factors such as protein binding, which may also play a significant role in how dosing decisions are made [8,12]. The technical aspects of how the Monte Carlo simulations were performed may also have an impact on PTA [13]. Given these potential complexities, we present simulations using a previously developed population PK model to highlight which clinical and technical factors most affect PTA. We also present a mini review of how authors used cefepime population PK models to perform PTA and to guide dosing decisions.

## 2. Results

### 2.1. Description of PTA Studies

The search term “probability of target attainment cefepime” yielded 49 results on 12JUL2022. The purpose of this mini review was to briefly survey the techniques and decisions researchers made while performing cefepime PTA. Therefore, only the first 20 titles were reviewed, of which 14 met the inclusion criteria of a PTA study, with at least one evaluation of cefepime alone in adult patients (Table 1) [14,15,16,17,18,19,20,21,22,23,24,25,26,27]. A study by Das et al. was excluded because cefepime was studied in combination with a novel beta-lactamase inhibitor, changing the risk benefit from cefepime alone [28]. A study conducted by Costenaro et al. was excluded because the it only included a pediatric population. Two studies from Jang et al. [29,30] were excluded because they used the same population PK model and similar decisions to a previous Jang et al. publication that was already included [15]. A study by Patel et al. was excluded because the authors did not perform their own PTA [31].

All authors reported a clear purpose for the PTA. Common purposes were to explore optimal doses in a special patient population, explore the success of achieving different PD targets, or exploring the success of one dosing regimen over another [14,15,17,18,25]. The number of virtual patients simulated varied from 1000 to 12,000. Some authors used an approach of simulating 1000 patients per subgroup of interest [18], while others simulated a large number such as 10,000 patients with the random selection of a demographic variable [24]. Typical values for the cefepime clearance (tvCL) were largely different across the studies (1.46–20.6 L/h), but they generally corresponded to the specific populations of interest in each study. For example, Sember et al. and Jang et al. used a tvCL of 1.46 L/h to perform simulations for patients assumed to be anuric receiving CKRT. Delattre et al. estimated a tvCL of 4.5 L/h for a more general critically ill population and was scaled to a CrCL of 100 mL/min. Álvarez et al. had an estimate of tvCL of 20.6 mL/min, scaled to a serum creatinine level of 0.46 mg/dL. Based on the demographic information presented by Álvarez et al., this approximately corresponds to a CrCl of 200 mL/min. Altough a CrCl of 200 mL/min is generally considered high, this may be explained by augmented renal clearance, which has been associated with febrile neutropenia [32,33,34].

Simulated dosing regimens most commonly consisted of 1–2 G q8 h infused over 30-min. Other commonly simulated dosing regimens were prolonged infusions of three or more hours, or continuous infusions with a total daily dose of 6000 mg or more of cefepime. PD targets of success were defined as fT 50–100% > 1–4 × MIC, generally with more stringent targets selected for critically ill populations [15,22,23]. The MIC of interest was generally set at 8 mg/dL, consistent with the breakpoint defined by the Clinical and Laboratory Standards Institute against pseudomonas aeruginosa [35]. Success was commonly defined as PTA > 90% against a given MIC; however, some authors defined success as CFR > 90% against an observed MIC distribution [18,20,21,26].

Pharmacokinetic parameters were most commonly simulated with one or two compartment PK models; however, two authors simulated the %fT based on equations [21,27]. Most authors did not specify the simulated time intervals. Of the authors that specified simulated time intervals, time-concentration points were simulated every 0.1 or 0.5 h [14,24]. The PK profiles were commonly simulated after the first dose, between the first 24 and 72 h or at steady state. A total of 6 G daily of cefepime was commonly recommended as either intermittent or continuous infusions. Delattre et al. recommended 4 G loading dose followed by 4 G q 6 h, which is markedly higher than the highest FDA labeled dose of 2 G q 8 h [36]. Several authors made no strong recommendation and took the approach of reporting PTA at accepted clinical doses [15,16,23].

### 2.2. Results of PTA Simulations

**Figure 1:**
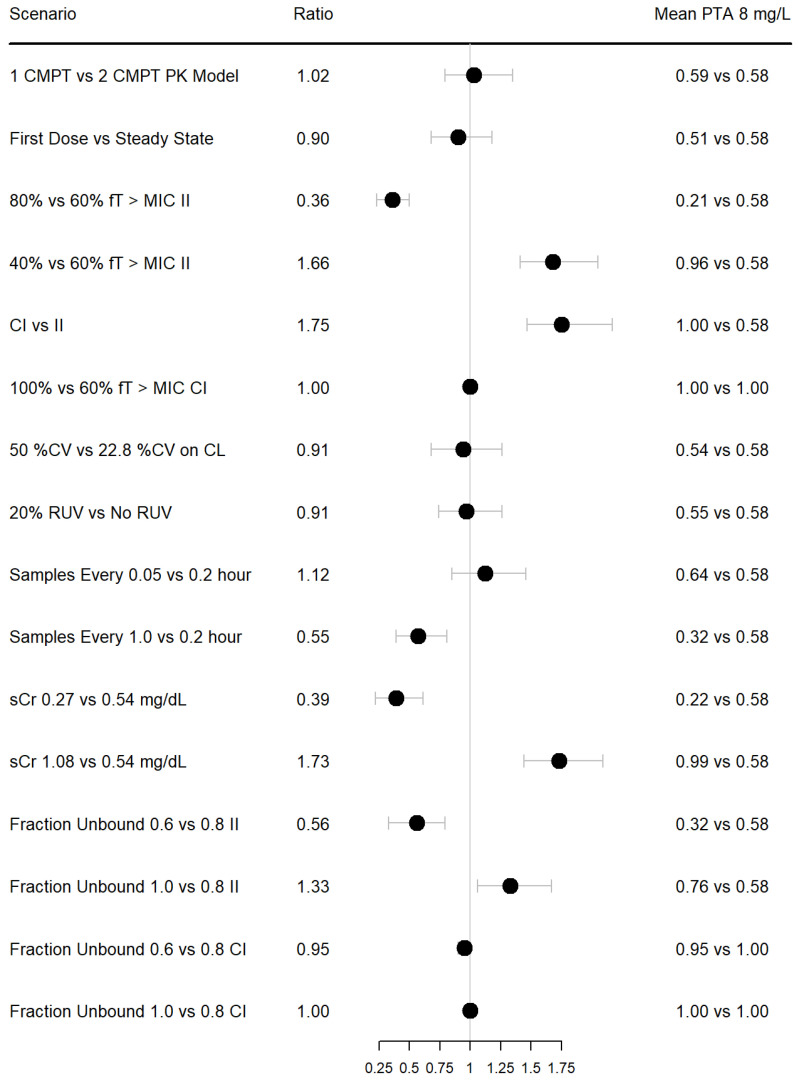
Forest plot of ratio of mean PTA under different simulation conditions using a population PK model developed by Álvarez et al. Dosing used was 2000 mg IV q8 h over 30 min unless otherwise stated. Continuous infusions matched the total daily dose of 6000 mg. Samples were simulated every 0.2 h unless otherwise stated. Error bars represent 90% CIs. II = intermittent infusion; CI = continuous infusion; RUV = residual unexplained variability; sCr = serum creatinine.

Common scenarios that possibly affect PTA results are summarized in Figure 1. All simulations were performed with the model and parameters derived from Álvarez et al., at an MIC of 8 mg/dL. Unless otherwise specified, the simulated dosing regimen was 2000 mg q8 h, infused over 30 min. Simulating with a one vs. two compartment PK model had minimal impact on the PTA (mean 59% vs. 58%). Similarly, simulating with high between-subject variability (50 CV) vs. observed (22.8% CV) and simulating with or without RUV had minimal impact on the PTA.

Simulating first dose vs. steady state demonstrated an average absolute 7% decrease in mean PTA; however, the 90% CI of the ratio of PTAs included 1. This is consistent with the short half-life of cefepime in patients with normal or augmented renal function [37]. This result may or may not be impactful, depending on how much a simulated dosing regimen supersedes the PTA, and may differ significantly in patients with impaired kidney function. Similarly, shortening the time interval of the simulated time-concentration sampling scheme (simulating every 0.05 vs. 0.2 h) was associated with an absolute increase in mean PTA by 6%, which may lead to different dosing conclusions if the threshold of success is achieved because of the simulated sampling scheme.

When considering intermittent infusion, halving the sCR (i.e., significantly increasing CrCl), using a PD target of fT 80% > MIC compared to fT 60% > MIC, simulating time-concentration observations every 1 vs. 0.2 h, and assuming 60% fraction unbound vs. 80% fraction unbound, significantly lowered the mean PTA by absolute 36%, 37%, 26% and 26%, respectively. The largest increases in mean PTA when considering intermittent infusion was under the conditions of doubling the sCR (i.e significantly reducing CrCl), using a PD target of fT 40% > MIC compared to fT 60% > MIC and assuming 100% fraction unbound vs. 80% fraction unbound, which were associated with absolute increases of 41%, 38% and 18%, respectively. However, when simulating with continuous infusions of 6000 mg/day, these different scenarios had minimal effect on mean PTA, which all approached 100%.

## 3. Discussion

We have presented a mini review of how researchers previously used cefepime PTA to guide dose optimization, in addition to simulations demonstrating the effects of different simulation conditions on mean PTA. This work builds upon the efforts of Mouton et al. [8], who proposed the standardization of PKPD terminology for anti-infectives, and the work of Kidd et al., who demonstrated that different technical methods of PTA may significantly change the result for beta-lactams [13]. Our main focus was on clinical factors that may affect PTA; however, we also presented some technical scenarios.

Our results highlight that although PTA is a powerful tool, the result of the PTA must be taken in the context of the patient population, and appropriate demographics must be input into the model for the simulation of covariate effects on PTA. For example, Álvarez et al. used a tvCL corresponding to an sCr of 0.47 mg/dL, which corresponds to a CrCl of approximately 200 mL/min. However, the mean reported CrCl by Álvarez et al. was 129 mL/min. This discrepancy may be explained because Álvarez et al. studied cefepime PK in patients with hematologic malignancy, who may have been cachectic and produced less daily sCr due to decreased muscle mass. Therefore, although the model itself was well validated, the use of sCr may have led to an overestimate of the tvCL for the purpose of PTA in this population. Using an sCr of 0.7 mg/dL (CrCl of approximately 130 mL/min, given the mean demographics) would have produced a tvCL of 11.1 L/h compared to 13.6 L/h. The careful selection of an exponential covariate model may have minimized the bias of PTA results in the case of Álvarez et al.; however, this highlights the importance of choosing clinically appropriate covariates relevant to the specific patient population.

Selecting appropriate dosing regimens for simulations is also essential for making appropriate recommendations. Lau et al. concluded that patients with a lower CrCl are at risk for cefepime underexposure. However, this is counterintuitive as cefepime is cleared by the kidneys and CrCl or sCr are commonly used covariates for explaining variability in cefepime CL. Rather, this may simply be a reflection of the dosing regimens selected, as the authors noted “a wide range of renal function defined by the >50 mL/min category, potentially resulting in overdosing when the same dosage is used, and the dose reduction in impaired renal function may be overly conservative.”

The optimal PD target for cefepime and beta-lactams in general is currently debated and may differ among various patient populations [7,38]. Achieving the optimal PD target appeared to be the primary driver of dosing recommendations from studies in the mini review. There is a strong rationale for this approach as sub-optimal antibiotic exposure may be associated with increased mortality [39]. However, as the optimal exposure for cefepime may be defined as fT 50–100% > 1–8XMIC, dose recommendation may differ significantly based on the chosen PD target. For example, Delattre et al. assumed a PD target of fT 70% > 4XMIC and recommended 4 G loading dose of cefepime infused over 3 h, followed by 4 G q 6 h. Huang et al. assumed a PD target of fT 50% > MIC and recommended 2 G q8 h. Although there are signals that achieving higher PD targets may be associated with improved clinical outcomes, these signals are often derived from secondary analyses and may be prone to bias [40]. Furthermore, cefepime is associated with possible neurotoxicity at exposure levels of 60 mg/L [41]. Although it was likely considered by all studies, only 3 of the 14 studies in the mini review performed a formal toxicodynamic analysis for cefepime [14,16,22]. Given the ongoing research and literature discussions about optimal beta-lactam PD targets and the risk for neurotoxicity, the final cefepime dosing recommendations should be based on a multi-disciplinary approach, considering the PTA, toxicity and the specific clinical and resource setting.

A final consideration is the use of PTA against a static MIC target vs. a CFR against an observed MIC distribution. For a given patient population, selecting PTA against a static breakpoint will be a more conservative PD metric and generally lead to higher dosing recommendations as compared to CFR. This is because CFR better captures the probability of achieving successful exposure across the MIC distribution of a specific patient population (including bacteria with lower MICs). This is highlighted by Liu et al., who found that no simulated dosing regimen met the 90% threshold for MIC > 8 mg/L in patients with CrCl > 60. In contrast, CFR > 90% was met for cefepime 2 G q8 h, based on MIC distributions from the SENTRY database [18,42]. However, it is important to note that some patient populations have higher rates of resistance, so PTA and CFR may not be comparable across different patient populations. For example, Thompson et al. reported MIC_50_ = 16 mg/dL and MIC_90_ = 512 mg/dL against pseudomonas aeruginosa, based on 43 isolates sampled from patients with cystic fibrosis [27]. Overall, both the PTA and CFR are reasonable PD metrics. The choice of PTA or CFR and subsequent dosing recommendations should, again, be based on the specific clinical setting as well as the resources available, with multi-disciplinary input when possible.

Limitations to this study include simulations with a single antibiotic, using a single-population PK model, and including only a limited number of studies in the mini review. However, the studies included were from the years 2016 to 2022, representing the modern applications of PTA. The studies generally utilized similar PTA techniques applied to sufficiently different patient populations. Therefore, although the possibility of selection bias remains, the included articles appropriately demonstrate the successes and pitfalls of modern PTA applications. Nevertheless, the results of this research should not be considered broadly applicable to all antibiotics, which may have different PD metrics altogether. Further, this research may not apply to all beta-lactams that have different physiochemical properties, leading to differences in PK, protein binding and the ability to kill bacteria. However, this study highlights several important factors when interpreting or performing PTA for the purpose of dose optimization, and the general principles apply to all beta-lactam antibiotics. Although challenging, further research such as randomized controlled trials testing different PD targets, with outcomes such as clinical cure rates and mortality, may help to better define the optimal PD targets and further improve PTA analyses.

## 4. Materials and Methods

PubMed was searched using the string “probability of target attainment cefepime” and yielded 49 results on 12JUL2022. The purpose of this mini review was exploratory; therefore, only the first 20 titles were reviewed. Cefepime was chosen because it is an FDA-approved broad-spectrum beta-lactam antibiotic for the treatment of a multitude of common bacterial infections in diverse populations. As such, cefepime is a reasonable representative of the beta-lactam class. Furthermore, the literature search yielded a sufficient number of studies to explore differences in how cefepime PTA was used for dose optimization. Studies were included if they contained a cefepime PTA or CFR analysis in an adult patient population. Pediatric studies and studies of the same author using the same population PK model as a previous publication were excluded. Given the exploratory nature of this mini review, we wanted to capture as many methods and techniques as possible, rather than highlight repeated techniques of a single author to answer different dose optimization questions.

Simulations were performed using Pumas v 1.1 [43]. A population PK model developed by Álvarez et al. was used to simulate individual PK profiles [17]. This model was chosen because the PTA results were reproducible and parameter estimates were ideal for demonstrating how PTA results may change, with different assumptions in technique or clinical covariates. For each simulation scenario (Figure 1), 50 patients were simulated, with the simulation repeated 200 times. A PTA was generated for each repetition in each scenario by calculating the percentage of patients achieving fT > MIC = 8 mg/L. The mean PTA was calculated as the average of the PTA from the 200 repetitions. The ratio of PTAs for each repetition were also calculated. From this dataset of the ratios of PTAs, the mean ratio and empiric 90% confidence intervals were calculated and plotted, using R (version 4.2.2) and R Studio (version 2022.07.2 Build 576) with the “forestplot” package. Simulated dosing regimens were either 2000 mg q8 h, infused over 30 min, or a 6000 mg/day continuous infusion.

## Figures and Tables

**Table 1:** Summary of studies included in mini review of using PTA to optimize cefepime dosing.

Study	Simulated Population	tvCL in Model	Simulated Doses	PD Target and Definition of Success	TD Target	Assumed Fraction Unbound	Simulation of PK Parameters	Simulated Time Intervals	Final Dose Recommendation
Álvarez	2500 patients with hematologic malignancy	13.6 L/h scaled to sCR of 0.47	4–8 G/day as 30-min infusions, extended infusions or continuous infusions	fT 60% or 100% > MIC = 4 mg/L or 8 mg/L. PTA 90%	Toxicity not analyzed	0.8, fixed	Pop PK model developed from plasma samples of 15 patients	Not specified, first 24 h and at steady state	2 G q 8 with extended 4-h infusions achieves lenient PD target and 6 g/day CI achieves all targets.
Butterfield-Cowper	5000 patients	5.43 L/h scaled to CrCl of 80 mL/min	30-min and 5-min infusions of 1 G q 6 h and 2 G q 8 h	70% fT 70% > 1 × MIC at hour 24 of therapy	Toxicity not analyzed	0.8, fixed	2-cmpt pop PK model developed by Tam et al.	Not specified	Minimal difference in PK profile from 30-min to. 5-min infusion.
Chaijamorn	5000 anuric CKRT patients	1.46 L/h (patients assumed to be anuric)	1 to 2 g every 12 h to 2 g loading dose followed by 1 g every 8 h or 2 g every 12 h	≥70% fT > 4 × MIC = 8 mg/L in a 48-h time period. PTA 90%	Probability of trough >= 70 mg/L at end of 48-h interval	0.79, simulated with mean and SD	Log-normal distribution based on 1-compartment PK model developed via a literature search	Not specified, initial 48 h	1.75–2 G loading dose followed by 1.5–2 G q 8 h.
Delattre	1000 patients per group	4.5 L/h scaled to CrCl of 100 mL/min and weight of 70 kg	4 g or 6 g administered as a 0.5-h, 2-h or 3-h infusion every 8 h	70%T > 4 × MIC ≤8 mg/L within a dosing interval. PTA 90%	Toxicity not analyzed	1, fixed	Pop PK developed from 88 critically ill patients	Not stated	4 G loading dose infused over 3 h followed by 4 G q 6 h.
Huang	10,000 healthy patients	5.3 L/h corresponds to CrCl of 100–120 mL/min (need to follow up on Nye et al.)	1 g every 12 h (q 12 h), 1 g every 8 h (q 8 h), 2 g q 12 h, and 2 g q 8 h as an IV bolus (an assumption for the equation to generate %fT > MIC)	fT 50% ≥ MIC based on observed MIC distribution and 90% CFR defined as success	Toxicity not analyzed	0.8–0.9 uniformly distributed.	CL estimated from a study of healthy volunteers Nye et al., %fT generated from an equation.	Equation used, steady state	2 G q 8 h IV bolus PTA achieved > 90% to MIC 16 mg/L; however, adequate for non-esbls, not adequate for esbl based on CFR
Jang	10,000 patients receiving CRRT	1.46 L/h (assumed to be anuric)	Cefepime 1 and 2-g q 8 or q 12 h over 30-min infusion	fT 60% ≥ MIC of 8 mg/L (also 4XMIC tested) in 72-h time period. PTA 90%	Toxicity not analyzed	0.79, fixed	Log-normal distribution based on 1-compartment PK model developed via a literature search	Not stated, 72 h of initial therapy	No dose recommendation, 2 G q 8 achieved > 90% PTA in all simulated subgroups.
Lau	12,000 patients	2.29 L/h scaled to CrCl 60 mL/min with linear model	Per Australian dosing guidelines	Cmin > 32 mg/L. PTA 90%	49 mg/L derived via ROC analysis	Not specified, assumed to be 1	Via population PK model developed by Jonckheere et al.	Cmin at steady state	No recommendation, 89% of patients with CrCl > 50 mL/min would achieve PTA dosing of 2 G q 8 h.
Liu	1000 patients per group, fixed at 70 kg and varied CrCl	5.65 L/h scaled to CrCL 120 mL/min and 70 kg	1–2 G q 8–12 as 2-, 5- or 30-min infusions	70%fT > MIC. CFR based on SENTRY database of MIC distributions. PTA 90%, CFR 90%	Toxicity not analyzed	0.8, fixed	Pop PK model developed from 70 patients and 604 cefepime concentrations	Not specified, evaluated 1st dose	IVP is not likely to be as good as intermittent infusion. No regimen meets the 90% threshold for MIC > 8 mg/L in patients with CrCl > 60, but CFR is > 90% for 2 G q 8 h based on MIC distributions.
Koomanachai	5000 patients	6.04 L/h scaled to CrCl 103.74 mL/min per equation in Tam et al.	2 g every 12 h (0.5-h infusion) or 2 g every 8 h (0.5-h and 3-h infusion)	≥50% fT > MIC. CFR >= 90% against observed MIC distribution	Toxicity not analyzed	Not stated	Simulated used Tam et al.	Not stated, steady state	2 g q 8 h infused over 3 h achieved CFR > 80–90%
Rhodes	10,000 patients with CrCl simulated range 108–220 mL/min	6.33 L/h scaled to CrCl of 120 m/min	3–8 G/day infused over 0.5–24 h q6–12 h or CI	≥68% fT > 1 × MIC in first 24 h of therapy. PTA 90%	Toxicity not analyzed	0.8, fixed	2-cmpt Pop PK model developed via cefepime concentration data from 9 patients	Simulated every 0.5 h, first 24 h of therapy	3–4 g/day as continuous infusions and doses of 2 g administered q 6 h (0.5-h infusion) to q 8 h (2-h infusion)
Sember	5000 anuric patients receiving CRRT	1.46 L/h (patients assumed to be anuric)	2-g loading dose (LD) infused over 0.5 h, followed by 1 or 2-g every 8 or 12 h with a 4-h extended-infusion.	≥60% fT > 4 × MIC = 8 mg/L in a 48-h time period. PTA 90%	Probability of trough >= 20 mg/L at end of 48-h interval	0.79, fixed	Log-normal distribution based on 1-compartment PK model developed via a literature search	Every 0.1 h for initial 48 h	2 G load followed by 2 G q 8 h
Shaw	5000 anuric patients receiving CRRT	1.49 L/h (patients assumed to be anuric)	1 to 2 g every 8–12 h to 2 g with or without load 2 G loading dose	≥60% fT > 1 × MIC or 4 × MIC = 8 mg/L in a 48-h time period. PTA 90%	Toxicity not analyzed	0.79, fixed	Log-normal distribution based on 1-compartment PK model developed via a literature search	Not specified, initial 72 h	No recommendation, but 2 G q 12 achieved 100% PTA in lenient target and 88.58% in strict target.
Thompson	10,000 patients with Cystic Fibrosis	8.47 L/h scaled to CrCl of 111.11 mL/min	2 g every 8 h (bolus and prolonged infusion)	≥60% or 100% fT > MIC against observed MIC distribution in CF patients (MIC_50_ = 16 mg/L). PTA 90%.	Toxicity not analyzed	0.8, fixed	Simulated via equations using steady state CL from Huls et al.	Equations used N/A	2 G CI achieves 66% PTA success and therefore is not adequate to cover resistant pseudomonal strains in CF population
Wang	5000 patients with CrCl >= 50 mL/min	9.18 L/h, which scales to a CrCl of 166.25 mL/min as calculated from Nicasio’s equation for CL_T_ = 0.048 × CLCR + 1.2	1 g q 12 h or 2 g q 12 h as 30-min infusion or 2 g q 12 h as 3-h infusion	50% fT > MIC within dosing interval based on observed MIC distribution with CFR 90% defined as success	Toxicity not analyzed	0.85, fixed	Used Pop PK developed by Nicasio et al.	Not stated, evaluated at steady state	2 g q 12 h, 3 h; and cefepime 2 g q 12 h, 0.5 h had CFR of 80–90% which was considered suboptimal and therefore other antibiotics were recommended.

## Data Availability

Data may be made available on reasonable request.
